# Supplementary material for: WordCommentsAnalyzer: A windows software tool for qualitative research
Source: F1000Res. 2018 Sep 4;7:536. Originally published 2018 May 3. [Version 2] doi: 10.12688/f1000research.14819.2 (PMC6137411; doi:10.12688/f1000research.14819.2)
Supplement: Supplementary file 3 [file f1000research-7-17614-s0002.tgz › b2a79496-4ada-47c4-9682-a99ce867836d.zip!/Eight Iranian Health Journals/Journal of Research in Rehabilitation Sciences _29.docx]

© Journal of Research in Rehabilitation Sciences . This work is licensed under CC BY-NC 4.0 (https://creativecommons.org/licenses/by-nc/4.0/).

Volume & Issue: دوره 9، شماره 4: 1392

Title: بررسی تاثیر روش‌های گفتاردرمانی همراه با درمان شناختی-رفتاری بر کاهش شدت لکنت افراد بالای 18 سال مبتلا به لکنت شهر اصفهان

http://jrrs.mui.ac.ir/index.php/jrrs/article/view/765

چکیده مقدمه: لکنت فقط یک مشکل گفتاری نیست. لکنت اختلالی است که سرتاسر جسم و روان فرد را دربرمی‌گیرد. برای رسیدن به درمان کامل بهتر است علاوه بر توجه به ناروانی‌های گفتار به جنبه‌های روانشناختی فرد هم توجه شود  و برای آن برنامه‌ریزی صورت گیرد. هدف از انجام این پژوهش بررسی تاثیر «گفتار درمانی همراه با درمان شناختی-رفتاری» بر کاهش شدت لکنت افراد بالای 18 سال مبتلا به لکنت در شهر اصفهان می‌باشد. مواد و روش‌ها: اين پژوهش از نوع نيمه‌آزمايشي و طرح پیش‌آزمون - پس‌آزمون با گروه کنترل است. گروه نمونه 24 نفر از افراد مبتلا به لکنت مراجعه‌کننده به کلینیک‌های دولتی گفتاردرمانی شهر اصفهان بودند. جهت تشخیص شدت لکنت در نمونه‌ها از آزمون شدت لکنت (SSI4) استفاده شد. آزمودنی‌ها پس از انجام پیش‌آزمون به صورت کاملا تصادفی در دو گروه آزمایش وکنترل قرار گرفتند. گروه آزمایش 10جلسه گفتاردرمانی و 10 جلسه درمان شناختی-رفتاری دریافت کرد. سپس، از هر دو گروه پس‌آزمون به عمل آمد. جهت تجزيه و تحليل آماري، از آزمون تحلیل کوواریانس و آزمون غیرپارامتریک یومان‌ویتنی استفاده گردید. یافته‌ها: نتایج تحلیل داده‌ها نشان داد که گفتاردرمانی همراه با درمان شناختی-رفتاری بر کاهش شدت لکنت تأثیر معنی‌داری داشته است(001/0= p). نتیجه‌گیری: گفتاردرمانی همراه با درمان شناختی-رفتاری بر کاهش شدت لکنت موثر است.

Title: بررسی تکرارپذیری ارزیابی خطای بازسازی نیرو در عضلات اکستانسور آرنج مردان جوان سالم

http://jrrs.mui.ac.ir/index.php/jrrs/article/view/834

چکیده مقدمه:حس عمقی از آن جهت که در مقابل صدمات حرکتی محافظ خوبی بوده و حفظ کننده ثبات مفاصل و هماهنگ کننده طبیعی آنها در هنگام حرکت است حائز اهمیت می باشد. از آنجا که بازسازی حس نیرو یکی از روش های رایج برای ارزیابی حس عمقی است ، ضروری است تا تکرارپذیری آن مورد بررسی قرار گیرد.بنابراین هدف از انجام مطالعه حاضر بررسی تکرارپذیری اندازه گیری خطای بازسازی حس نیرو در عضلات اکستانسور آرنج اندام غالب و مغلوب ،در مردان جوان سالم می باشد. مواد و روش ها:در این مطالعه12 مرد جوان سالم در محدوده سنی 18 تا 30 سال شرکت داشتند ، که به روش نمونه گیری غیرتصادفی ساده در دسترس از میان دانشجویان دانشکده توانبخشی دانشگاه علوم پزشکی شهید بهشتی در سال 1391 انتخاب شدند.تکرارپذیریIntra-testerو Inter-day مورد بررسی قرار گرفت.برای ارزیابی خطای بازسازی حس نیروی عضلات اکستانسور آرنج با استفاده از نیروسنج، درصد مشخصی (30٪) از حداکثر انقباض ایزومتریک را به فرد آموزش داده و از او خواستیم نیروی آموزش داده شده را بازسازی نماید.پس از 2 دقیقه از فرد خواسته می شد تا مجددا نیروی هدف را بازسازی نماید. آزمون در هر مرحله سه بار تکرار می شد.خطای حس نیروی فرد با محاسبه میانگین تفاوت میزان نیروی هدف و نیروی ساخته شده در طی سه تکرار برای بار اول و دوم محاسبه شد.مطالعه در دو مرحله به صورت تصادفی در اندام غالب و مغلوب انجام گردید.برای بررسی تکرارپذیری از روش تکرارپذیری نسبی  (ICC)، تکرارپذیری مطلق (SEM) ومفایسه مقادیر دو تکرار با آزمون تی زوجی   استفاده شد. یافته ها:مقادیر ICC برای خطای ثابت، مطلق و متغیر بازسازی حس نیرو در اندام غالب (راست) و مغلوب (چپ) از 76/0 تا 94/0 بود که تکرارپذیری بالایی را نشان می دهد. مقادیر SEM از 004/0تا 02/0 بود. نتیجه گیری:بر اساس یافته های این مطالعه ، نتیجه گرفته شد که روش ارزیابی خطای حس نیرو در هر دو اندام در  مردان جوان سالم از تکرارپذیری بالایی برخوردار است و می توان از آن به عنوان یک روش پایا برای ارزیابی خطای بازسازی نیرو استفاده کرد.

Title: بررسی اثر انواع بریس بر روی شاخص‌های کنترل قامت در ورزشکاران دارای مچ پای ناپایدار درجه یک و دو

http://jrrs.mui.ac.ir/index.php/jrrs/article/view/587

چکیده مقدمه: هدف از این تحقیق بررسی اثر سه نوع بریس) نئوپرینی ،ایرکست و بنددار) بر روی شاخص‌های کنترل قامت در والیبالیست‌های دارای مچ پای ناپایدار درجه یک و دو می‌باشد. مواد و روش‌ها:  آزمودنی‌های این تحقیق 15 والیبالیست در دامنه سنی 18 تا 25 سال باحداقل 3 سال سابقۀ بازی و دارای مچ پای ناپایدار درجه 1 و2 بودند. در طول مراحل تحقیق  چهار بار(بدون بریس، بریس نئوپرینی، بریس بنددار، و بریس ایرکست) آزمون کنترل قامت توسط دستگاه تعادلی بایودکس (تست کنترل قامت ورزشکاران بر روی یک پا در سطح دشواری12) صورت گرفت. داده‌های تحقیق با استفاده از روش آماری آنالیز اندازه‌های مکرر یکطرفه تجزیه و تحلیل شد و سطح معنی داری 95/0 با آلفای کوچکتر یا مساوی 05/0استفاده گردید. يافته‌ها: نتایج نشان داد که میزان شاخص کلی ثبات هنگام بکارگیری هر سه نوع بریس در مقایسه با شرایط عدم استفاده از بریس کاهش معنی‌داری دارد، یا به عبارتی هر سه نوع بریس باعث بهبود کنترل قامت می‌شوند. هنگام مقایسه سه نوع بریس، بریس نئوپرینی بهتر از دو نوع بریس دیگر عمل کرد. ولی در مقایسۀ دو نوع بریس بنددار و ایرکست اختلاف معنی‌داری بین این دو مشاهده نشد. به عبارتی در بهبود کنترل قامت، بریس نئوپرینی بهتر از بریس ینددار و ایرکست عمل می‌کند. اگرچه بریس بنددار تا حدودی بهتر از ایرکست عمل می‌کند، اما این دو نوع بریس در بهبود کنترل قامت اختلاف معنیدار با همدیگر ندارند.  نتيجه‌گيری: با استفاده از  یافته‌های موجود می­توان از بریس به عنوان روشی مناسب جهت بهبود کنترل قامت در افراد دارای مچ پای ناپایدار و جهت پیشگیری از آسیب‌های لیگامانی استفاده کرد و برای افراد دارای ناپایداری درجه یک و دو استفاده از بریس نئوپرینی می‌تواند نتایج بهتری نسبت به استفاده از دو نوع بریس دیگر داشته باشد.

Title: مقایسه ادراک شنیداری کودکان کاشت حلزون شده، دارای سمعک و کودکان با شنوایی طبیعی

http://jrrs.mui.ac.ir/index.php/jrrs/article/view/1035

چكيده مقدمه: آسیب شنوایی تمام جنبه‌های زندگی فرد، به ویژه ادراک شنیداری را تحت تأثیر قرار می‌دهد، در حالی که استفاده از کاشت حلزون یا سمعک، تأثیر مطلوبی بر  عملکرد شنیداری دارد. هدف این پژوهش، مقایسه ادراک شنیداری کودکان کاشت حلزون شده، دارای سمعک و کودکان با شنوایی طبیعی در شهرستان‌های تهران بود. مواد و روش‌ها: در این پژوهش تحليلي و مقايسه‌اي 60 پسر 5 تا 7 سال شرکت کرده بودند. آنها به سه گروه 20 نفری تقسیم شدند به نحوی که گروه اول و دوم به ترتیب از کودکان کاشت حلزون شده و دارای سمعک بودند که به روش نمونه‌گیری در دسترس انتخاب شده بودند؛ در حالی که گروه سوم، شنوایی طبیعی داشتند و به روش تصادفی انتخاب شده بودند. ابزارهاي استفاده شده در اين پژوهش آزمون هوشی Raven و مقیاس تعیین سطح عملکرد شنیداری بود. اطلاعات جمع‌آوری شده با استفاده از تحلیل واریانس یک طرفه و آزمون تعقیبی Scheffe در نرم‌افزار SPSS نسخه 16 تحلیل شد. یافته‌ها: اختلاف ميانگين ادراک شنیداری بين گروه‌هاي مورد مطالعه معنی‌دار بود (0005/0P< و 99/42= (57 و 2)F). میانگین نمرات ادراک شنیداری کودکان با شنوایی طبیعی به طور معنی‌داری بیشتر از دو گروه دیگر بود (0005/0P<). همچنین میانگین نمرات ادراک شنیداری کودکان کاشت شده به طور معنی‌داری بیشتر از کودکان دارای سمعک بود  (002/0P<). نتیجه‌گیری: کودکان با شنوایی طبیعی، ادراک شنیداری بهتری از کودکان کاشت حلزون شده و دارای سمعک داشتند ولی از آن جایی که استفاده از کاشت حلزون و سمعک نیز منجر به بهبود ادراک شنیداری کودکان ناشنوا شد. بنابراین به برنامه توانبخشی ویژه‌ای نیاز است تا تحول بیشتری در مهارت ادراک شنیداری کودکان کاشت حلزون شده یا دارای سمعک ایجاد شود.

Title: مقایسه شیوع، نوع و شدت آسیب ‌در کشتیگیران حرفه‌ای و آماتور آزادکار

http://jrrs.mui.ac.ir/index.php/jrrs/article/view/866

چكيده مقدمه:محققان تلاش می‌کنند که تا با بررسی میزان شیوع آسیب در ورزش‌های مختلف دلایل آن‌ها را مشخص و در نهایت راهکارهای پیشگیری مناسبی را برای آن‌ها ارائه کنند. با این حال هنوز هم آسیب‌های زیادی در ورزش‌ها مشاهده شده و الگوهای آسیب‌دیدگی در ورزشکاران رشته‌های مختلف ورزشی نامشخص باقی مانده است. هدف از مطالعه حاضر مقایسه شیوع، مکانیسم، نوع و شدت آسیب‌های حاد در کشتی‌گیران حرفه‌ای و آماتور آزادکار بود. مواد و روش‌ها:بدین منظور 28 کشتی‌گیر حرفه‌ای، به صورت در دسترس هدف‌دار و 28 کشتی‌گیر آماتور به صورت خوشه‌ای تصادفی از سه استان همدان، کرمانشاه و کردستان به عنوان آزمودنی‌های تحقیق انتخاب شدند. ابزار جمع آوری اطلاعات،‌ پرسش‌نامه محقق ساخته‌ای بود که توسط محقق تدوین و توسط متخصصان تأیید شده بود. این پرسش‌نامه به منظور ارزیابی شدت، شیوع، نوع و سازوکار آسیب کشتی‌گیر در یک سال گذشته توسط کشتی‌گیران تکمیل شد. اطلاعات جمع‌آوری شده به وسیله نرم‌افزار آماری SPSSنسخه 16 و روش‌های آماری توصیفی و استنباطی خی دو در سطح معناداری 5% >p تجزیه و تحلیل شد. يافته‌ها:یافته‌های تحقیق حاضر حاکی از آن بود که تنها بین نوع آسیب‌دیدگی کشتی‌گیران آماتور و حرفه‌ای آزادکار تفاوت معنی‌داری وجود دارد (019/0p=)، و در هر دو گروه کشتی‌گیران، سهم آسیب‌های شدید بیشترین و شیوع آسیب در حین تمرینات مخصوص کشتی بیشتر (کشتی‌گیران آماتور 3/70% و حرفه‌ای 3/64%) بود. علاوه بر این معمول‌ترین مکانیسم آسیب‌دیدگی برای کشتی‌گیران حرفه‌ای خطای حریف (0/30%) و برای کشتی‌گیران آماتور نیز عدم آمادگی جسمانی (8/37%) و خطای حریف (1/31%) گزارش شد. شایع‌ترین ناحیه آسیب‌پذیر نیز توسط هر دو گروه سر، صورت و گردن، شانه و زانو گزارش شد. نتيجه‌گيري: با وجود این‌که فرض بر آن بود که با توجه به متفاوت بودن سطوح آمادگی جسمانی، تجهیزات و تجربیات کشتی‌گیران حرفه‌ای و آماتور آزادکار، گستره‌ وقوع آسیب‌ها در سطح حرفه‌ای و آماتور متفاوت باشد، اما این‌چنین نبود؛ لذا برای اظهارنظر قطعی لزوم انجام تحقیق در سطح گستر ده‌تر است.

Title: تعيين میزان آگاهی و استفاده مراقبین از شیوه شیوه های مراقبت ويژه کودکان 5-1 ساله فلج مغزی

http://jrrs.mui.ac.ir/index.php/jrrs/article/view/848

مقدمه: هدف از مطالعه حاضر بررسی میزان آگاهی و استفاده مراقبین از شیوه‌های مراقبت ويژه کودکان فلج مغزی 5-1 سالهبود. مواد و روش‌ها:اين مطالعه به صورت مقطعي، توصيفي_تحليلي انجام گرفت. براي جمع آوري داده‌ها، از پرسشنامه‌اي پژوهشگر ساخته که اعتبار آن در شش مرحله مورد ارزیابی و ارتقاء قرار گرفته بود، استفاده شدو 70 نفر از مراقبین مراجعه‌کننده به مراكز توانبخشي شهر تهران در اين تحقيق شركت نمودند. داده‌ها‌ با استفاده از آمار توصيفي و ضريب همبستگي اسپيرمن در نرم‌افزار آماریSPSS نسخه 16تحليل و بررسي گرديد. يافته‌ها:سنجش ميزان آگاهي براساس خوداظهاري نشان داد که 1/87 % از مراقبین آگاهي خود را در حد ضعیف و متوسط تخمين زده بودند. سنجش ميزان آگاهي براساس آزمون نشان داد که 3/94 % از مراقبین دارای آگاهي ضعیف و متوسط بودند. همچنین نتایج نشان داد که 1/27 % از افراد شرکت‌کننده در پژوهش در زمینه مراقبت ویژه از کودکان فلج مغزی آموزش ندیده‌اند. همه افراد آموزش ديده به يك نسبت تمامي حيطه‌ها را آموزش نديدهبودند. بررسی سوابق آموزشی آنان نشان داد که بیشترین زمینه آموزش دیده شده تحرک و جابجایی )1/57%) و كمترين در زمينه دستشويي گرفتن (0/20%) بود. نتيجه‌گيري: آگاهي اكثريت افراد شركت كننده در این پژوهش ضعیف و متوسط اندازه‌گیری گردید. اين يافته ها ضرورت بازنگري در شیوه های آموزشی مراقبت‌های ویژه كودكان فلج مغزی به مراقبین را بيان مي‌دارد. بعلاوه مراقبین نيز نيازمند شركت در كارگاه‌هاي آموزشي و دریافت جزوه‌های آموزشی مناسب می‌باشند تا ميزان آگاهي خود را افزايش دهند.بیشترین تأکید کارگاه‌های آموزشی باید بر آموزش روش‌های دستشویی گرفتن و تحرک و جابجایی باشد.

Title: تعیین همبستگی بین رشد مهارت های زبانی و میزان پرخاشگری رابطه ای در کودکان فارسی زبان 6-4 ساله شهر شیراز

http://jrrs.mui.ac.ir/index.php/jrrs/article/view/978

چكيده مقدمه: رشد زبان و رفتار اجتماعی از جنبه‌های مهم رشد اولیه کودکان محسوب می‌شوند. يکی از شایع‌ترین مشکلات رفتاری در دوران کودکی پرخاشگری رابطه‌ای، یعنی تلاش برای صدمه زدن به دیگران از طریق آسیب زدن به روابط اجتماعی است. پرخاشگری رابطه‌ای با عوامل مختلفی از جمله رشد زبان در ارتباط است که در این مطالعه به بررسی آن در کودکان ایرانی طبیعی پرداخته شد. مواد و روش⁯ها: تحقیق حاضر به روش غیرتجربی و مقطعی انجام شد. تعداد 106 کودک (64 پسر و 42 دختر) 4 تا 6 ساله که معیارهای ورود به مطالعه را داشتند، به روش تصادفی چندمرحله‌ای، از بین مهدهای کودک شهر شیراز انتخاب و با استفاده از نسخه فارسی آزمون رشد زبان (TOLD-P3) و پرسش‌نامۀ پرخاشگری رابطه‌ای ارزيابی شدند. جهت تعیین نوع و میزان همبستگی بین مهارت‌های زبانی و پرخاشگری رابطه‌ای در این کودکان، از ضریب همبستگی پیرسون و آزمون تی ـ مستقل در سطح معنی¬داری 05/0 استفاده شد. یافته‌ها: رشد زبان و پرخاشگری رابطه‌ای دارای رابطۀ مثبت و معنی‌دار بودند (01/0>p). به عبارت دیگر، با افزایش مهارت‌های زبان درکی و بیانی در کودکان پیش‌دبستانی، احتمال بروز پرخاشگری رابطه‌ای در آن‌ها وجود دارد. از سوی دیگر، میزان بروز پرخاشگری رابطه‌ای و رشد مهارت‌های زبانی در دختران به طور معنی‌داری بیشتر از پسران بود (01/0>p). نتیجه‌گیری: مهارت‌های زبان درکی و زبان بیانی بهتر در پسران 6-4 ساله می‌تواند اثرات تسهیل‌کننده در بروز رفتارهای پرخاشگرانه رابطه‌ای داشته باشد. در صورتی‌که نمی‌توان چنین نقشی را برای رشد زبان در دختران 6-4 ساله قائل شد.

Title: مقایسه ی تأثیر فوری ارتوز AFO مرسوم و ارتوز لگني- مچ پايي بر پارامترهای راه رفتن در افراد مبتلا به بیماری ام اس

http://jrrs.mui.ac.ir/index.php/jrrs/article/view/1001

چکیده مقدمه: بیماری ام اس، بیماری خودایمنی سیستم اعصاب مرکزی است. در اغلب موارد در اثر این بیماری، عصبهای نخاعی و عصبهای بینایی آسیب می‌بینند. درمان ارتوزی در این بیماران، با توجه به ضعف عضلات مچ و هیپ و به دنبال افتادگی مچ پا بصورت متداول استفاده از AFO می باشد. بازخورد برخی از بیماران و همینطور نظر دست اندرکاران حاکی از آنست که بسیاری از بیماران از ارتوز AFO استفاده نمی نمایند. از طرف دیگر ارتوزی به نام لگنی- مچ پایی با ادعای کاهش مشکلات این گروه از بیماران در منابع طرح شده که در ایران تاکنون ساخته نشده است. مواد و روش ها: در این مطالعه 17 فرد مبتلا به بیماری ام اس با درگیری یکطرفه در اندام تحتانی شرکت کردند. پارامتر های راه رفتن شامل سرعت و تقارن گام پس از آزمون 2 دقیقه ای راه رفتن در حالت بدون ارتوز و با ارتوز AFOمرسوم و ارتوز لگنی- مچ پایی محاسبه گردید. تأثیر شاخص جثه، میزان اثربخشی ارتوز و رضایت از طریق پرسشنامه نیز در این افراد مورد بررسی قرار گرفت. یافته ها: نتایج آزمون t زوج نشان می دهد، سرعت راه رفتن و تقارن گام در هنگام استفاده از ارتوز لگنی – مچ پایی در مقایسه با ارتوز AFO مرسوم دارای اختلاف معنادار می باشدP<0.001)). همچنین میزان رضایت از ارتوز AFO مرسوم با ارتوز لگنی-مچ پایی نیز دارای اختلاف معنادار است. در ارتباط با تاثیر شاخص جثه با اثربخشی مداخلات انجام شده بر روی سرعت و تقارن گام، اختلاف آماری معناداری مشاهده نگردید. نتیجه گیری: نتایج مطالعه حاضر نشان می دهد که ارتوز لگنی– مچ پایی بصورت آنی، در بیماران ام اس با ضعف عضلات مچ پا و هیپ سبب بهبود در تقارن گام و افزایش سرعت راه رفتن می گردد. همینطور به نظر می رسد شاخص جثه عامل اثرگذاری در تجویز این ارتوز نمی باشد.

Title: بررسی ارتباط بین ویزگی های سایکواکوستیک وزوز و میزان معلولیت ناشی از آن در کارگران شرکت ذوب آهن اصفهان

http://jrrs.mui.ac.ir/index.php/jrrs/article/view/1006

چكيده مقدمه:قرار گیری در معرض نویز یکی از علل شناخته شده در ایجاد وزوز است. تعیین ویژگی‌های سایکواکوستیکی وزوز به تنهایی نمی‌تواند ارزیابی دقیقی از وزوز و تأثیر آن بر روی کیفیت زندگی باشد. هدف از این مطالعه بررسی ارتباط ویژگی‌های سایکواکوستیک وزوز با میزان معلولیت ناشی از آن است. مواد و روش‌ها:مطالعه حاضر از نوع توصیفی-تحلیلی است. افراد شرکت‌کننده 20 کارگر شاغل در محیط صنعتی و دارای کم شنوایی و وزوز بودند. بعد از در نظر گرفتن معیارهای ورود، ارزیابی‌های سایکواکوستیکی انجام و پرسش‌نامه THIتکمیل گردید. يافته‌ها:میانگین و انحراف معیار THIبرابر 8/25±7/53 به دست آمد. نتایج نشان داد که بین مدت زمان ابتلا به وزوز و بلندی وزوز با امتیازات THIارتباط مثبت معنی‌داری وجود دارد (p ˂0.05). بین PTA، SDSو دیگرپارامترهای سایکواکوستیکی وزوز با THIارتباط معنی‌داری یافت نشد. نتيجه‌گيري: ارریابی های سایکواکوستیکی به تنهایی نمی توانند شاخص معتبری از میزان تاثیر وزوز بر کیفیت زندگی باشند. استفاده همزمان از آزمون های سایکواکوستیکی و پرسشنامه ها برای ارزیابی های وزوز معتبرتر هستند.

Title: اثر بخشی خدمات کاردرمانی مرکز روزانه بر عزت نفس و طول مدت بستری بیماران مبتلا به اختلال مزمن روان

http://jrrs.mui.ac.ir/index.php/jrrs/article/view/914

چکیده مقدمه: عزت نفس پایین به طور مستمر با اختلالات روانپزشکی مرتبط است. کاردرمانی به افزایش عزت نفس از طریق انجام فعالیت‌های هدفمند می‌پردازد و عمیقاً به رابطه کار و سلامتی معتقد است. در پژوهش حاضر به بررسی اثر بخشی خدمات کاردرمانی مرکز روزانه بر عزت نفس و طول مدت بستری بیماران مزمن روان پرداخته شده است. مواد و روش­ها: این مطالعه مداخله‌ای به صورت شبه تجربی می‌باشد که طی آن 20 بیمار مبتلا به اختلالات مزمن روان، خدمات کاردرمانی مرکز روزانه بیمارستان روانپزشکی 505 را دریافت کردند، به عنوان گروه آزمایش در نظر گرفته شدند و 20 بیمار دارای اختلال مزمن روان که این خدمات را دریافت نمی‌کردند، در گروه کنترل قرار گرفتند. نمونه‌ها از طریق نمونه گیری در دسترس انتخاب شدند. مداخله کاردرمانی مرکز روزانه، 2 روز در هفته، هر روز 5 ساعت به مدت 3 ماه و 20 جلسه، برگزار شد. شرکت‌کنندگان مقیاس عزت نفس کوپر اسمیت را برای ارزیابی میزان عزت نفس تکمیل کردند. میانگین روزهای بستری یکسال قبل و یک‌سال بعد از انجام مداخله محاسبه شد. جهت تحلیل داده‌ها از نرم‌افزار آماری  SPSS نسخه 19، استفاده گردید. یافته­ها: نمرات عزت نفس بیماران بعد از انجام مداخلات کاردرمانی افزایش معنی‌داری داشت(04/0 =P).همچنین، طول مدت بستری بیماران پس از مداخله کاهش یافت(03/0 =P). نتیجه­گیری: استفاده از خدمات کاردرمانی به صورت مرکز روزانه می‌تواند به افزایش عزت نفس و کاهش طول مدت بستری مبتلا به بیماران اختلالات مزمن روان مؤثر باشد.

Title: اثر خستگی عضلات مرکزی بدن بر آزمونهای عملکردی اندام تحتانی در مردان ورزشکار

http://jrrs.mui.ac.ir/index.php/jrrs/article/view/916

چکیده مقدمه: ثبات مرکزی بدن توانایی کنترل موقعیت و حرکت تنه بر روی لگن و اندام تحتانی جهت تولید و انتقال مثر نیرو به بخش‌های انتهایی بدن در طول فعالیت‌های ورزشی است. نقش ثبات مرکزی بدن در عملکرد اندام تحتانی در تعدادی از مطالعات مورد ارزیابی قرار گرفته است، اما نتایج این‌گونه تحقیقات با یکدیگر همسو نیستند. در این زمینه، نشان داده شده است که بررسی اثر خستگی عضلات مرکزی بدن بر عملکرد اندام تحتانی می‌تواند اطلاعات مفیدی را درباره نقش ثبات مرکزی بدن در عملکرد اندام تحتانی فراهم آورد. بنابراین هدف از انجام این مطالعه بررسی اثر خستگی عضلات مرکزی بدن بر آزمون‌های عملکردی اندام تحتانی در مردان ورزشکار بود. مواد و روش‌ها: 34 دانشجوی مرد سالم رشته تربیت بدنی و علوم ورزشی در یک جلسه آزمون‌های عملکردی اندام تحتانی شامل آزمون‌های تعادلY، جهش جانبی، جهش هشت لاتین و سه جهش تک‌پا را در شرایط عادی (بدون خستگی) انجام دادند. در جلسه دوم، ابتدا ورزشکاران در پروتکل خستگی عضلات مرکزی بدن که شامل هفت تمرین بود شرکت کرده و بلافاصله آزمون‌های عملکردی اندام تحتانی را انجام دادند. به منظور مقایسه نتایج آزمون‌های عملکردی اندام تحتانی قبل و بعد از خستگی از آزمون آماری تی زوجی استفاده شد. یافته‌ها: پروتکل خستگی عضلات مرکزی بدن موجب کاهش معنی‌داری در نمرات تمامی آزمون‌های عملکردی اندام تحتانی شامل آزمون‌های تعادلY، جهش جانبی، جهش هشت لاتین و سه جهش تک‌پا شد (001/0P = برای تمامی آزمون‌ها). نتیجه‌گیری: از نتایج این تحقیق می‌توان استنباط کرد که کارکرد عضلات مرکزی بدن بر عملکرد اندام تحتانی تأثیر می‌گذارد زیرا خستگی عضلات مرکزی بدن موجب کاهش نمرات تمامی آزمون‌های عملکردی اندام تحتانی در این مطالعه گشت. با توجه به احتمال خسته شدن عضلات مرکزی بدن طی فعالیت‌های بلند مدت ورزشی، تمریناتی که باعث افزایش استقامت عضلات مرکزی بدن و بهبود توانایی آن‌ها در مقابله با خستگی می‌شوند، احتمالاً می‌توانند به عنوان راهی برای جلوگیری از کاهش عملکرد اندام تحتانی طی فعالیت‌های طولانی مدت ورزشی پیشنهاد شوند.

Title: بررسی تأثیر تحریک دهانی پیش از تغذیه به روش بکمن بر عملکرد تغذیه‌ای نوزادان نارس

http://jrrs.mui.ac.ir/index.php/jrrs/article/view/952

چکیده مقدمه: هدف این مقاله بررسی تأثیر برنامه تحریک دهانی پیش از تغذیه بکمن بر زمان دستیابی به تغذیه دهانی کامل نوزادان نارس 32-26 هفته جنینی بستری در واحد مراقبت‌های ویژه نوزادان بیمارستان کمالی کرج می‌باشد. مواد و روش‌ها: مطالعه از نوع کارآزمایی بالینی و تصادفی است و جامعه مورد مطالعه، نوزادان نارس با سن جنینی 26-32 هفته (21 نوزاد)، بستری در واحد مراقبت‌های ویژه نوزادان بیمارستان کمالی کرج می‌باشند. بعد از اعمال معیار ورود و خروج، نوزادان به دو گروه، «تحریک دهانی پیش از تغذیه بکمن» و گروه کنترل تقسیم شدند. برنامه‌ی تحریک دهانی پیش از تغذیه با توجه به حساسیت بالای کار و اهمیت استمرار انجام آن، در 10 روز متوالی بدون وقفه برای هر نوزاد توسط پژوهشگر انجام شد. آزمودنی‌های گروه کنترل و آزمایش از لحاظ سن تقویمی و سن جنینی در دفعات مختلف تغذیه دهانی و در زمان ترخیص از واحد مراقبت های ویژه نوزادان و میزان وزن‌گیری در هفته اول و دوم تولد و زمان ترخیص مورد بررسی و مقایسه قرارگرفتند. يافته­ها: در گروه بکمن، میانگین سن تقویمی در زمان 8 بار تغذیه دهانی، 18/9 ±20/18 روز (میانگین ± انحراف معیار) بوده است و در گروه کنترل،42/10 ± 27/24 روز بوده است؛ در نتیجه گروه آزمایش با تفاوت 07/6 روز زودتر به این معیار دست یافتند که این عدد به لحاظ بالینی بسیار اهمیت دارد. در گروه بکمن، میانگین سن تقویمی در زمان ترخیص از بیمارستان 57/12 ±60/27 روز بوده است و در گروه کنترل، 46/13 ± 45/33 روز بوده است که از نظر میانگین، گروه آزمایش 85/5 روز زودتر از بیمارستان ترخیص گردیدند که از لحاظ بالینی و اقتصادی اهمیت فراوانی دارد. نتیجه‌گیری: این مطالعه، از مطالعات بیشتر در زمینه تحریک دهانی بکمن به منظور افزایش مهارت تغذیه دهانی و کاهش مدت بستری در بیمارستان حمایت می­کند.

Title: برداشت انرژی از راه رفتن جهت تامین انرژی یک سیستم الکترونیکی با استفاده از مبدل پیزوالکتریک

http://jrrs.mui.ac.ir/index.php/jrrs/article/view/999

چکيده مقدمه: اگرچه با کاهش ابعاد و مصرف مدارهای الکترونیکی، امکان توسعه بسیاری از ابزارهای پزشکی به صورت قابل پوشیدن و یا نصب­شونده در داخل بدن فراهم شده است، اما وزن و اندازه بزرگ باتری مورد نیاز برای تأمین انرژی ابزار، باعث محدود بودن کاربرد این ابزارها و یا مشکلات در استفاده درازمدت از آن‌ها ­است. رویکرد نوین برای رفع این محدودیت، کسب انرژی لازم برای عملکرد دستگاه، از خود بدن می­باشد. مواد و روش ­ها: در این مقاله، توسعه سیستمی برای کسب انرژی از راه رفتن با استفاده از مبدل پیزوالکتریک با هدف تأمین انرژی یک ابزار پزشکی پوشیدنی ارائه شده است. مبدل پیزوالکتریک درون کفش زیر پاشنه پا کار گذاشته شده است و انرژی کسب شده در هر گام، ذخیره و برای استفاده ابزارهای الکترونیکی مناسب­سازی شده است. سپس با انجام آزمایش­های عملی کارآیی این ابزار مورد ارزیابی قرار گرفته است. یافته­ ها: ابزاری که در این تحقیق توسعه داده شد، امکان کسب و ذخیره انرژی فشار پا بر زمین حین راه رفتن را دارا است. این ابزار با استفاده از تنها یک مبدل پیزوالکتریک، امکان تأمین توان دایمی در حد 8/0 میلی­وات را فراهم نمود. البته برای فراهم شدن این توان دایمی به صورت یک منبع تغذیه 3 ولت، نیاز به برداشته شدن حدود 40 گام به صورت اولیه بود. این ابزار جهت تأمین انرژی یک مدار الکترونیکی پایه مورد استفاده قرار گرفت و پایداری آن در طول زمان مورد تأیید قرار گرفت. نتیجه­ گیری: با توسعه ابزارهای پزشکی پوشیدنی کم مصرف، امکان تأمین انرژی لازم برای آن‌ها از خود بدن و جایگزین شدن این روش بجای استفاده از باتری وجود دارد.

Title: بررسی تأثیر معنی کلمه بر بسامد ناروانی گفتار در بزرگسالان دارای لکنت رشدی

http://jrrs.mui.ac.ir/index.php/jrrs/article/view/1077

مقدمه: لکنت از شایع­ترین اختلالات گفتار و زبان است. سبب­شناسی لکنت از جنبه­های گوناگونی مانند؛ زیستی، رشدی، عاطفی، یادگیری و زبان­شناختی مورد بررسی قرار گرفته است. تحقیقات نشان می­دهند برخی ویژگی­های زبان­شناختی مانند معنی کلمه می­تواند بسامد وقوع ناروانی در این اختلال را تحت تأثیر قرار دهد. بنابراین پژوهش حاضر با هدف تعیین تأثیر معنی کلمه بر بسامد ناروانی گفتار در بزرگسالان دارای لکنت رشدی انجام شده است. مواد و روش­ها: پژوهش توصیفی- تحلیلی حاضر به صورت مقطعی بر روی 14 فرد بزرگسال دارای لکنت با میانگین سنی 25 سال انجام شده است. بسامد ناروانی­های گفتار از طریق خواندن دو فهرست؛ شامل 60 کلمه و 60 شبه­کلمه مورد ارزیابی قرار گرفت. کلمه­ها بر اساس ساختارهای هجایی رایج در زبان فارسی انتخاب شدند. اطلاعات به دست آمده توسط نرم افزار SPSS و با استفاده از آزمون آماري Paired t-test  مورد تجزيه و تحليل قرار گرفت. یافته­ها: نتایج بررسی نشان می­دهد، تفاوت معنی­داری بین میانگین بسامد ناروانی­های گفتار در فهرست کلمه­ها و شبه­کلمه­ها وجود دارد(05/0>P). نتیجه­گیری: این یافته آشکار می­سازد، بسامد ناروانی گفتار در شبه­کلمه­ها به میزان قابل توجهی بیش از کلمه­ها است. بنابراین به نظر می­رسد به دلیل حذف بازیابی محتوایی معنایی در تکلیف خواندن شبه کلمه ها، فرایند رمزگذاری واجشناختی در تکلیف خواندن شبه­کلمه­ها بسیار پیچیده­تر از تکلیف خواندن کلمه­ها باشد.

Title: ارتباط بین سطح فعالیت برخی از عضلات اندام تحتانی با نیروی برشی قدامی زانو در حرکت پرش-فرود تک‌پا

http://jrrs.mui.ac.ir/index.php/jrrs/article/view/1133

چکیده  مقدمه: شناسایی فاکتورهایی که باعث شیوع بالای آسیب ACL می‌شود از اهمیت زیادی برخوردار است. نیروهای برشی قدامی مکانیزم اصلی بار وارده به ACL هستند. فعالیت عضلات و پوزیشن اندام تحتانی طی فعالیت می‌تواند باعث افزایش یا کاهش نیروی برشی قدامی شود. هدف از این مطالعه بررسی ارتباط بین سطح فعالیت برخی از عضلات اندام تحتانی با نیروی برشی قدامی در حرکت پرش-فرود تک‌پا بود. مواد و روش‌ها: 20 دانشجوي پسر رشته تربیت بدنی در اين مطالعه شركت كردند. سطح فعالیت الکترومیوگرافی عضلات سرینی میانی، ساقی قدامی و همسترینگ خارجی با استفاده از دستگاه الکترومیوگرافی، و نیروی عکس‌العمل زمین با استفاده از دستگاه صفحه نيرو در حركت پرش- فرود تک پا ارزیابی شد. به منظور تجزیه و تحلیل داده­ها، از آزمون همبستگی پیرسون استفاده شد. یافته‌ها: نتایج آماری نشان داد که بین سطح فعالیت عضله ساقی قدامی و سرینی میانی با نیروی برشی قدامی ارتباط معنی داری وجود ندارد (05/0P>)، اما ارتباط معنی داری بین سطح فعالیت عضله همسترینگ خارجی با نیروی برشی قدامی یافت شد (05/0P<). نتیجه‌گیری: یافته­های این مطالعه نشان داد که فعالیت عضله همسترینگ در حین حرکت پرش- فرود تک‌پا به عنوان عضله حمایت کننده از ACL عمل می‌کند و افزایش فعالیت این عضله می‌تواند منجر به کاهش بار وارده بر ACL شود.

Title: مقایسه نتایج آزمون فاصله در نویز در افراد بزرگسال با شنوایی هنجار و مبتلا به کم شنوایی انتقالی

http://jrrs.mui.ac.ir/index.php/jrrs/article/view/910

مقدمه: کم شنوایی انتقالی سبب کاهش و تأخیر، انتقال صدا در گوش میانی می­شود. این مسئله می­تواند انواعی از اختلالات در عملکرد شنوایی مرکزی را ایجاد کند که حتی پس از درمان مشکلات محیطی همچنان باقی می­ماند. یکی از جنبه­های پردازش شنوایی مرکزی، پردازش زمانی است که نقش مهمی در درک گفتار دارد و شامل تفکیک، ترتیب، تجمع و پوشش زمانی است. از آنجا که کم شنوایی انتقالی می­تواند سبب کاهش توانایی تفکیک زمانی شود؛ در این مطالعه به منظور بررسی اثر کم شنوایی انتقالی بر توانایی تفکیک زمانی از آزمون فاصله در نویز استفاده شده است. مواد و روش­ها: پژوهش توصیفی-تحلیلی غیر مداخله ای حاضر روی 13 فرد با کم شنوایی انتقالی دو طرفه مختصر تا متوسط و 13 فرد با شنوایی هنجار در محدوده سنی 50-20 سال با شرایط جنسی و سنی مشابه انجام گرفت. سپس شاخص­های مورد بررسی شامل آستانه تقریبی و درصد پاسخ­های صحیح آزمون فاصله در نویز با آزمون­های من­ویتنی، ویلکاکسن بین دو گروه مقایسه شد. یافته­ها: اثری از جنسیت و گوش آزمایشی بر نتایج آستانه تقریبی و درصد پاسخ­های صحیح در دو گروه دیده نشد (05/0p>) در حالی که گروه مبتلا به کم شنوایی انتقالی آستانه تقریبی بالاتر و درصد پاسخ­های صحیح کمتری نسبت به گروه با شنوایی هنجار کسب کردند (001/0p<). نتیجه­گیری: افزایش آستانه تشخیص فاصله و کاهش درصد پاسخ­های صحیح در گروه مبتلا به کم شنوایی انتقالی نشان دهنده اثر کم شنوایی انتقالی بر فعالیت بخشی از مسیر سیستم شنوایی مرکزی و کاهش توانایی تفکیک زمانی شنوایی است.

Title: مقایسه رضایت شغلی در گفتار درمان‌گران و کاردرمان‌گران کرج

http://jrrs.mui.ac.ir/index.php/jrrs/article/view/1446

چكيده مقدمه: رضایت شغلی از عوامل مهم افزایش بهره وری، تعلق و دلبستگی کارکنان به محیط کار و افزایش کمیت و کیفیت کار است. بنابراین بررسی این عامل در مشاغل مختلف اهمیت بسیاری دارد. مواد و روش‌ها: این بررسی از نوع توصیفی – تحلیلی می باشد. مطالعه در بین کاردرمان‌گران و گفتاردرمان‌گران شهرستان کرج انجام گرفته است. از هر گروه 16 نفر به صورت تصادفی پرسشنامه دموگرافیک و پرسشنامه رضایت شغلی (JDI) را تکمیل نمودند. روایی و پایایی این پرسشنامه در تحقیقات مشابه داخلی مورد تایید بوده است. یافته ها در نرم افزار SPSS و با آزمونهای ناپارامتریک آنالیز گردید. يافته‌ها: نمرات رضایت شغلی در کاردرمان‌گران (76/0±31/3) بیشتر از گفتاردرمان‌گران (45/0±80/2) به دست آمد. در گروه گفتاردرمان‌گران بین رضایت شغلی با سرپرستی، حقوق و ترفیع رابطه معنی دار به دست آمد. در کاردرمان‌گران رضایت شغلی با ماهیت کار، حقوق، ترفیع و همکاران رابطه معنی دار نشان داد. نتيجه‌گيري: نمرات به دست آمده در هر دو گروه نشانگر انست که اقدامات اصلاحی ضروری می باشد. اقداماتی برای افزایش امنیت شغلی، بهبود شرایط ارگونومی محیط کار، توسعه شغلی و غنای شغلی می تواند به افزایش رضایت شغلی در این دو گروه کمک نماید.

Title: بررسی و مقایسه ی زمان واکنش آواسازی افراد دارای لکنت و بدون لکنت در دو گروه سنی 20-10 و30-20 سال

http://jrrs.mui.ac.ir/index.php/jrrs/article/view/873

چکیده مقدمه: گروهی از محققین لکنت را اختلال در زمان‌بندی بین عناصر آوایی و وقفه در برنامه‌ریزی هم‌زمان و موفق حرکات عضلات گفتاری جهت تولید یک کلمه می‌دانند. براون و همکاران پی بردند که حرکات گفتاری افراد مبتلا به لکنت از نظر زمان‌بندی در مقایسه با افراد بدون لکنت متفاوت است؛ از جمله‌ی این متغیرهای زمانی می‌توان به زمان واکنش اشاره کرد. زمان واکنش معیاری است که به طور دقیق می‌تواند ماهیت تفاوت‌های آواسازی بین افراد مبتلا به لکنت و بدون لکنت را نشان دهد. هدف این مطالعه بررسی و مقایسه‌ی زمان واکنش آواسازی افراد مبتلا به لکنت و بدون لکنت می‌باشد. مواد و روش‌ها: این مطالعه از نوع توصیفی- تحلیلی بوده و در آن 22 نفر از افراد دارای لکنت و 22نفر بدون لکنت از سنین 10 تا 30 سال انتخاب شدند و از آنها خواسته شد که به محض شنیدن محرک، پاسخ مورد نظر (کشیدن صدای a) را ارائه دهند. در ادامه هر دو  محرک و پاسخ  توسط نرم افزار Praat ضبط و فاصله‌ی بین آنها اندازه‌گیری شد. یافته‌ها‌: این پژوهش نشان داد که میانگین زمان واکنش افراد مبتلا به لکنت بیشتر از افراد بدون لکنت بوده و این اختلاف به خصوص در گروه سنی 20 تا 30 سال بارزتر بود. این در حالی است که اختلاف زمان واکنش در دو گروه سنی معنادار نبود. بحث: با توجه به وجود مشکل در شروع آواسازی در افراد مبتلا به لکنت می‌توان چنین نتیجه گرفت که این مسأله می‌تواند نتیجه‌ی مشکلاتی از قبیل تنش عضلانی و رفتارهای اجتنابی، مشکلات مربوط به فاکتورهای عصبی- حرکتی و یا فشار زمان احساس شده توسط فرد مبتلا به لکنت بخصوص در افراد بزرگسال که تجربهی بیشتری در لکنت دارند، باشد.

Title: بررسی تأثیر معنی کلمه بر بسامد ناروانی¬ گفتار در بزرگسالان دارای لکنت رشدی

http://jrrs.mui.ac.ir/index.php/jrrs/article/view/1084

چکیده مقدمه: لکنت از شایع­ترین اختلالات گفتار و زبان است. سبب­شناسی لکنت از جنبه­های گوناگونی مانند؛ زیستی، رشدی، عاطفی، یادگیری و زبان­شناختی مورد بررسی قرار گرفته است. تحقیقات نشان می­دهند برخی ویژگی­های زبان­شناختی مانند معنی کلمه می­توانند بسامد وقوع ناروانی گفتار در این اختلال را تحت تأثیر قرار دهند. بنابراین پژوهش حاضر با هدف تعیین تأثیر معنی کلمه بر بسامد ناروانی گفتار در بزرگسالان دارای لکنت رشدی انجام شده است. مواد و روش ­ها: پژوهش توصیفی- تحلیلی حاضر به صورت مقطعی بر روی 14 فرد بزرگسال دارای لکنت (12 مرد و 2 زن) با میانگین سنی 93/24 سال انجام شده است. بسامد ناروانی­های گفتار از طریق خواندن دو فهرست؛ شامل 60 کلمه و 60 شبه­کلمه مورد ارزیابی قرار گرفت. کلمه­ها بر اساس ساختارهای هجایی متنوع در زبان فارسی انتخاب شدند. اطلاعات به دست آمده توسط نرم افزار SPSS و با استفاده از آزمون آماري Paired t-test  مورد تجزيه و تحليل قرار گرفتند. یافته­ها: نتایج بررسی نشان داد، تفاوت معنی­داری بین میانگین بسامد ناروانی­های گفتار در فهرست کلمه­ها و شبه­کلمه­ها وجود دارد (05/0>P). نتیجه­گیری: این یافته آشکار می­سازد، بسامد ناروانی گفتار در شبه­کلمه­ها به میزان قابل توجهی بیش از کلمه­ها است. بنابراین به نظر می­رسد به دلیل حذف بازیابی محتوایی معنایی در تکلیف خواندن شبه­کلمه­ها، فرایند رمزگذاری واجشناختی در تکلیف خواندن شبه­کلمه­ها بسیار پیچیده­تر از تکلیف خواندن کلمه­ها باشد.

Title: مقایسه¬ی تأثیر فوری ارتوز AFO مرسوم و ارتوز لگني- مچ پايي بر پارامترهای راه رفتن در افراد مبتلا به بیماری ام اس

http://jrrs.mui.ac.ir/index.php/jrrs/article/view/1026

چکیده مقدمه: بیماری ام اس، بیماری خودایمنی سیستم اعصاب مرکزی است. در اثر این بیماری، گاهی عصب-های نخاعی و عصب-های بینایی آسیب می‌بینند. درمان ارتوزی، با توجه به ضعف عضلات مچ و هیپ و به دنبال افتادگی مچ پا، بصورت متداول در ایران استفاده ازAFO  می-باشد. بنابر مطالعات، بسیاری از بیماران از ارتوز AFO استفاده نمی-نمایند. از طرف دیگر ارتوزی به نام لگنی- مچ پایی در تحقیقات با ادعای کاهش مشکلات این گروه از بیماران مطرح شده است. هدف از مطالعه حاضر مقایسه تأثیر فوری ارتوز AFO مرسوم و ارتوز لگنی- مچ پایی بر پارامترهای راه رفتن در افراد مبتلا به بیماری ام اس می-باشد. مواد و روش ­ها: در این مطالعه 17 فرد مبتلا به بیماری ام اس با درگیری یکطرفه در اندام تحتانی شرکت کردند. پارامتر های راه رفتن شامل سرعت و تقارن گام پس از آزمون 2 دقیقه-ای راه رفتن در حالت بدون ارتوز و با ارتوز  AFOمرسوم و ارتوز لگنی- مچ پایی محاسبه گردید. تأثیر شاخص جثه، میزان اثربخشی ارتوز و رضایت از طریق پرسشنامه نیز در این افراد مورد بررسی قرار گرفت. یافته‌ها‌: نتایج آزمونt  زوج نشان داد، سرعت راه رفتن و تقارن گام با ارتوز  AFOمرسوم و حالت بدون ارتوز دارای اختلاف معنادار نمی-باشد، در حالی­که هنگام استفاده از ارتوز لگنی- مچ پایی بطور معنادار بهبود می-یابد. همچنین میزان رضایت از ارتوز لگنی- مچ پایی بیشتر از ارتوز AFO مرسوم بود. در ارتباط با تأثیر شاخص جثه با اثربخشی مداخلات انجام شده بر روی سرعت و تقارن گام، اختلاف آماری معناداری مشاهده نگردید. نتیجه‌گیری: نتایج مطالعه حاضر نشان می­دهد که ارتوز لگنی– مچ پایی بصورت آنی، در بیماران ام اس با ضعف عضلات مچ پا و هیپ سبب بهبود در تقارن گام و افزایش سرعت راه رفتن می­گردد. همینطور به نظر می­رسد شاخص جثه عامل اثرگذاری در تجویز این ارتوز نمی­باشد.

Title: مقایسه رضایت شغلی در گفتاردرمانگران و کاردرمانگران کرج

http://jrrs.mui.ac.ir/index.php/jrrs/article/view/792

مقدمه: رضایت شغلی از عوامل مهم افزایش بهره وری، تعلق و دلبستگی کارکنان به محیط کار و افزایش کمیت و کیفیت کار است. بنابراین بررسی این عامل در مشاغل مختلف اهمیت بسیاری دارد. مواد و روشها: این بررسی از نوع توصیفی – تحلیلی می باشد. مطالعه در بین کاردرمانگران و گفتاردرمانگران شهرستان کرج انجام گرفته است. از هر گروه 16 نفر به صورت تصادفی پرسشنامه دموگرافیک و پرسشنامه رضایت شغلی (JDI) را تکمیل نمودند. یافته ها در نرم افزار SPSS و با آزمونهای ناپارامتریک آنالیز گردید. یافته ها: نمرات رضایت شغلی در کاردرمانگران (76/0±31/3) بیشتر از گفتاردرمانگران (45/0±80/2) به دست آمد. در گروه گفتاردرمانگران بین رضایت شغلی با سرپرستی، حقوق و ترفیع رابطه معنی دار به دست آمد. در کاردرمانگران رضایت شغلی با ماهیت کار، حقوق، ترفیع و همکاران رابطه معنی دار نشان داد. نتیجه گیری: نمرات به دست آمده در هر دو گروه نشانگر انست که اقدامات اصلاحی ضروری می باشد. اقداماتی برای افزایش امنیت شغلی، بهبود شرایط ارگونومی محیط کار، توسعه شغلی و غنای شغلی می تواند به افزایش رضایت شغلی در این دو گروه کمک نماید.

Title: نرو-آکوپیشن: دیدگاه سامانه ی پویای غیرخطی به مکانیسم عصبی مغز

http://jrrs.mui.ac.ir/index.php/jrrs/article/view/1010

چکیدهمقدمه:«نرو-آکوپیشن» یکی از جدیدترین مدل های مطرح در کاردرمانی است. ماهیت این مدل بر اساس همزیستی و برهم کنش پویا بین عملکردهای سامانه ی عصبی مغز و اشتغال فرد در کار هایش استوار است. نرو-آکوپیشن دیدگاهی کل گرا و آمیزه ای از علم عصب شناسی و کار است. بر این اساس مغز انسان سامانه ی پیچیده ی سازش پذیر و خودساماندهی تلقی می گردد که از طریق کار های انجام گرفته توسط فرد و به هدف دستیابی به عملکرد ایده آل باید با محیط اطرافش در ارتباط باشد. در مقاله ی حاضر به تفصیل به معرفی این مدل پرداخته می شود. به جهت نشان دادن اینکه چگونه این مدل در مقایسه با سایر مدل های کاردرمانی، راهنمای ایجاد تفکری متفاوت نسبت به مراجعان است رفتارهای یک بیمار افسرده ی مبتلا به دیابت با استفاده از اصول آن آنالیز شده است.مواد و روش ها:یک جستجوی الکترونیک در پایگاه های اطلاعاتی بین المللی Google Scholar ، PubMed ، ISI web of knowledge و Scopus در بازه ی زمانی سالهای 1995 تا 2013 انجام گرفت. معیار انتخاب، محوریت متن هر منبع بر اساس عبارت نرو-آکوپیشن “Neuro-occupation” بود. بر این اساس تعداد 10 مقاله یافت شد.یافته ها:ساختار مدل نرو-آکوپیشن بر مبنای ارتباط تنگاتنگی است که بین فرآیندهای نرولوژیکی و کار فرض شده است. اجزای مدل شامل سه سطح «خواسته»، «معنا» و «درک» نشان می دهند که چگونه کارکرد سامانه ی عصبی مغز از طریق یافتن معنا در حین مشارکت فرد در کار های هدفمند منجر به ساماندهی رفتار وی به شکل بروز رفتارهایی انطباقی می شود بطوریکه او را قادر به مقابله با چالشهای زندگی می کند.نتیجه گیری: مدل نرو-آکوپیشن برای قشر گسترده ای از بیماران با تشخیص های متنوع کارآیی دارد. بنابراین با در نظر گرفتن پدیده ی خودساماندهی در کارکردهای سامانه ی عصبی مغز می توان از مراجع انتظار داشت که از مراحل «خواسته»، «معنا» و «درک» گذر کند.

Title: کاردرمانی مراجع‌محور مبتنی بر روش چهارچوب فرآیند کاری کانادایی در بیمار اسکیزوافکتیو: مطالعه موردی

http://jrrs.mui.ac.ir/index.php/jrrs/article/view/934

چکیده مقدمه: هدف از این مطالعه بررسی تأثیر مدل کاری مراجع‌محور در درمان مبتنی بر کار در بیمار اسکیزوافکتیو بر اساس چهارچوب فرآیندکاری کانادایی است. مواد و روش‌ها: در این تحقیق موردی، خانم 38 ساله با تشخیص اسکیزوافکتیو مورد مطالعه قرار گرفت. جهت ارزیابی از آزمون‌های مقیاس عملکرد کانادایی و مقیاس افسردگی بک 2 و همچنین آزمون شناختی لوتکا استفاده شد، مداخله به مدت 21 جلسه درمان مستقیم و در ادامه مشاوره مبتنی بر چهارچوب کاری عملکرد کانادایی انجام شد که درمان مراجع محور را تسهیل میکرد.در تمام فرآیند درمان ارزش‌ها و علایق مراجع مورد احترام قرار گرفت و مراجع در ارزیابی و تعیین اهداف، مشارکت فعال داشت. یافته‌ها:  نتایج بررسی میزان تأثیر مداخله با آزمون COPM حاکی از افزایش و تغییر میزان عملکرد و رضایت مراجع به میزان 7/5 و3/5 و کاهش شدت میزان افسردگی فرد ازشدید به متوسط(50 به 20) بود. نتیجه‌گیری: نتایج حاکی از موثر بودن این رویکرد در اختلال اسکیزوافکتیو به خصوص در حوزه  مهارت‌های اجتماعی و کارهای روزمره و کاهش افسردگی وی می‌باشد.
